# Expression of AMPA receptor subunits at synapses in laminae I–III of the rodent spinal dorsal horn

**DOI:** 10.1186/1744-8069-4-5

**Published:** 2008-01-23

**Authors:** Erika Polgár, Masahiko Watanabe, Bettina Hartmann, Seth GN Grant, Andrew J Todd

**Affiliations:** 1 Spinal Cord Group, Institute of Biomedical and Life Sciences, University of Glasgow, Glasgow G12 8QQ, UK; 2 Department of Anatomy, Hokkaido University School of Medicine, Sapporo 060-8638, Japan; 3 Institute for Pharmacology, University of Heidelberg, Im Neuenheimer Feld 366, 69120 Heidelberg, Germany; 4 Team 32: Genes to Cognition, Wellcome Trust Sanger Institute, Cambridge, UK

## Abstract

**Background:**

Glutamate receptors of the AMPA type (AMPArs) mediate fast excitatory transmission in the dorsal horn and are thought to underlie perception of both acute and chronic pain. They are tetrameric structures made up from 4 subunits (GluR1-4), and subunit composition determines properties of the receptor. Antigen retrieval with pepsin can be used to reveal the receptors with immunocytochemistry, and in this study we have investigated the subunit composition at synapses within laminae I–III of the dorsal horn. In addition, we have compared staining of AMPArs with that for PSD-95, a major constituent of glutamatergic synapses. We also examined tissue from knock-out mice to confirm the validity of the immunostaining.

**Results:**

As we have shown previously, virtually all AMPAr-immunoreactive puncta were immunostained for GluR2. In laminae I–II, ~65% were GluR1-positive and ~60% were GluR3-positive, while in lamina III the corresponding values were 34% (GluR1) and 80% (GluR3). Puncta stained with antibody against the C-terminus of GluR4 (which only detects the long form of this subunit) made up 23% of the AMPAr-containing puncta in lamina I, ~8% of those in lamina II and 46% of those in lamina III. Some overlap between GluR1 and GluR3 was seen in each region, but in lamina I GluR1 and GluR4 were present in largely non-overlapping populations. The GluR4 puncta often appeared to outline dendrites of individual neurons in the superficial laminae. Virtually all of the AMPAr-positive puncta were immunostained for PSD-95, and 98% of PSD-95 puncta contained AMPAr-immunoreactivity. Staining for GluR1, GluR2 and GluR3 was absent in sections from mice in which these subunits had been knocked out, while the punctate staining for PSD-95 was absent in mice with a mutation that prevents accumulation of PSD-95 at synapses.

**Conclusion:**

Our results suggest that virtually all glutamatergic synapses in laminae I–III of adult rat spinal cord contain AMPArs. They show that synapses in laminae I–II contain GluR2 together with GluR1 and/or GluR3, while the long form of GluR4 is restricted to specific neuronal populations, which may include some lamina I projection cells. They also provide further evidence that immunostaining for AMPAr subunits following antigen retrieval is a reliable method for detecting these receptors at glutamatergic synapses.

## Background

The superficial part of the spinal dorsal horn (laminae I–II) is the major target for nociceptive primary afferents [1-3]. It contains numerous excitatory and inhibitory interneurons, a population of projection cells that are located in lamina I, and the dorsally directed dendrites of neurons that have their cell bodies in laminae III and IV [4-7]. The circuitry of this region is complex and poorly understood, although it is known that many of these neurons respond to noxious stimulation [8-13] and that the projection cells appear to be necessary for the development of chronic pain states [14,15].

Glutamate is the main excitatory neurotransmitter in the dorsal horn, and is released by all classes of primary afferent, as well as by the axons of many spinal neurons and by certain axons that descend from the brain [16,17]. Glutamate acts on both ionotropic and metabotropic receptors, and these are widely expressed in the spinal cord [18]. In the dorsal horn, ionotropic receptors of the AMPA type (AMPArs) mediate fast EPSPs [19,20] and are thought to play a major role in the perception of both acute and chronic pain [21,22]. AMPArs have a tetrameric structure and are made up from four subunits (GluR1-4, also known as GluR-A-D) that are encoded by four separate genes, *gria1-4*. All four subunits are expressed in the dorsal horn [23-33]. Both homomeric and heteromeric receptors can be formed, and the properties of the receptors depend on subunit composition. AMPArs that lack the GluR2 subunit show significant Ca^2+^-permeability [34], while those that possess subunits with long C-terminal tails (GluR1 and GluR4) have been shown to undergo activity-dependent insertion, leading to long-term potentiation (LTP) [35]. In addition, the subunits have specific sites at which they can undergo phosphorylation, which results in alterations in the channel properties of the receptor [36]. We have previously demonstrated that acute noxious stimulation results in phosphorylation at the S845 site of GluR1 subunits at synapses in the superficial dorsal horn [33], and this is likely to lead to an increase in peak open probability of the receptors, and thus an enhancement of synaptic transmission [37].

Although there are specific antibodies directed against each of the AMPAr subunits, it is difficult to detect synaptic receptors with conventional immunocytochemistry, because the cross-linking of proteins in the synaptic cleft and post-synaptic density that occurs during fixation restricts the access of these antibodies in tissue sections. Antigen retrieval with pepsin [38] can be used to reveal synaptic receptors, and we have previously described the laminar distribution of GluR1-4 at synapses in the rat spinal cord [33]. We reported that GluR2 was widely distributed throughout the grey matter, and was apparently present in virtually all synapses that contained AMPArs, whereas GluR1 was largely restricted to laminae I–III of the dorsal horn. GluR3 and 4 were found at relatively high density in deep dorsal horn and ventral horn, but were also present in some synapses in the superficial laminae.

In this study we have used immunocytochemistry with antigen retrieval to provide detailed quantitative information about the expression of the different AMPAr subunits at glutamatergic synapses in laminae I–III of the dorsal horn, and to examine the pattern of co-expression of subunits at synapses in this region. In order to identify AMPAr-containing synapses, we used an antibody that recognises all four subunits (pan-AMPAr antibody) [39]. One of the main aims was to determine the proportion of synapses that contained GluR1 and/or GluR4, as this would give an indication of the extent to which synaptic plasticity involving these subunits could affect dorsal horn neurons. To provide further evidence that the immunostaining for AMPArs seen after antigen-retrieval with pepsin is located at glutamatergic synapses, we compared it with staining obtained with an antibody against the post-synaptic density protein PSD-95 [40]. In addition, we have used spinal cord sections from mice that lacked the genes for GluR1, 2 or 3 subunits [41,42] or expressed a mutant form of PSD-95 [43] to demonstrate specificity of synaptic labelling with the antibodies against these proteins.

## Results

### Distribution of AMPAr subunits in laminae I–III

**Figure 1:**
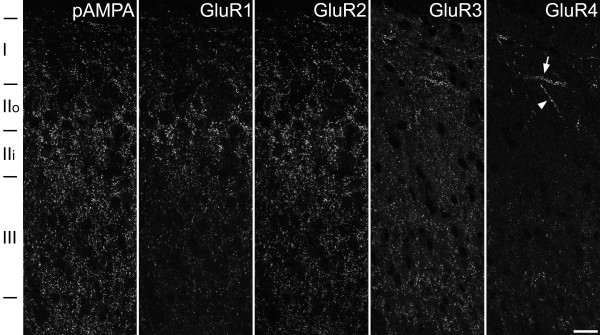
**Immunostaining with the pan-AMPAr antibody and with subunit-specific antibodies in laminae I–III of the rat dorsal horn following antigen retrieval with pepsin**. Each image shows a vertical strip taken through the central part of the dorsal horn stained with the pan-AMPAr antibody (pAMPA) or with one of the subunit-specific antibodies. As we have reported previously [60], lamina I is relatively thick in this region. Images of pan-AMPAr, GluR1 and GluR2 are taken from one section, while those for GluR3 and GluR4 are taken from another. GluR1 puncta are most numerous in laminae I and II, while those that are GluR2- or GluR3-immunoreactive are present in large numbers throughout laminae I–III. The GluR4 staining was obtained with a rabbit antibody against the C-terminal part of the protein (GluR4-C antibody), and is present at relatively few puncta in laminae I and II. However some clusters of GluR4 puncta that are orientated either transversely (arrow) or dorsoventrally (arrowhead) are visible in the superficial dorsal horn. Each image was obtained from a projection of 5 optical sections at 0.3 μm z-spacing. Approximate locations of laminar boundaries are shown. Scale bar = 20 μm.

**Figure 2:**
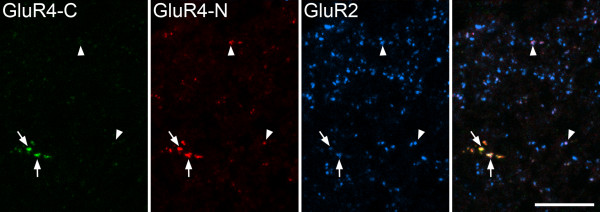
**Comparison of immunostaining with the GluR4-C and GluR4-N antibodies**. Confocal images from lamina I stained with the GluR4-C, GluR4-N and GluR2 antibodies. A few puncta are labelled with the GluR4-C antibody (two marked with arrows). The GluR4-N antibody stains these strongly, but also labels several other puncta more weakly (two shown with arrowheads). All of the puncta labelled by each GluR4 antibody are GluR2-immunoreactive. Images were obtained from 4 optical spacing at 0.3 μm. Scale bar = 10 μm.

Following antigen retrieval with pepsin, a punctate pattern of staining was seen in the dorsal horn with antibodies against each of the GluR subunits and also with the pan-AMPAr antibody (Fig. 1). The distribution of puncta seen with the GluR1, GluR2 and GluR3 antibodies was the same as that reported by Nagy et al. [33]. GluR1 puncta were largely restricted to laminae I–III of the dorsal horn, while GluR2 puncta were present throughout the grey matter, but were densest and most strongly stained in laminae I–II. GluR3-immunoreactive puncta were also present throughout the grey matter. The majority of those in laminae I and II were relatively weakly stained compared to puncta in deeper laminae, although scattered bright puncta were seen in lamina I. However, the rabbit GluR4 C-terminal antibody (GluR4-C) that was used in this study gave a different pattern of labelling to that previously observed in the dorsal horn with a guinea-pig antibody against the N-terminus of this subunit (GluR4-N) [33]. While the N-terminal antibody had labelled many puncta in the superficial laminae, the C-terminal antibody labelled few puncta, although these were often relatively bright and arranged in rows or clusters (Fig. 1 arrow, arrowhead). When we compared the staining with the 2 GluR4 antibodies directly, we found a population of puncta that were strongly stained with both the GluR4-N and GluR4-C antibodies, as well as many puncta that were more weakly stained with the GluR4-N antibody but were not labelled by GluR4-C. All of the puncta stained with either GluR4 antibody were also GluR2-immunoreactive (Fig. 2). The distribution of punctate staining with the pan-AMPAr antibody was very similar to that seen with the GluR2 antibody (Fig. 1).

### Quantification of AMPAr subunits

**Figure 3:**
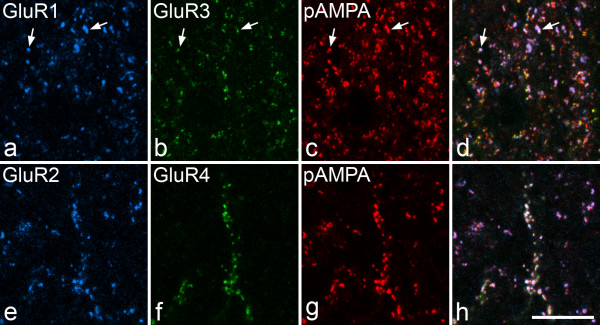
**Staining with GluR1-4 and pan-AMPAr antibodies**. Confocal images that show immunoreactivity for GluR1, GluR3 and pan-AMPAr (pAMPA) in lamina I (**a**-**d**), and GluR2, GluR4 and pan-AMPAr in lamina III (**e**-**h**). In each case a merged image is shown (**d**,**h**). Note that all puncta labelled with the GluR1-4 antibodies are also labelled with the pan-AMPAr antibody. **a**-**d**: Although most of the puncta that are strongly labelled with the GluR1 or GluR3 antibody are weakly labelled or negative with the other one, some can be seen to contain immunostaining for both subunits (2 shown with arrows). **e**-**f**: All of the puncta that are GluR4-positive are also labelled with the GluR2 and pan-AMPAr antibodies. The images are projections of 2 optical sections at 0.35 μm z-spacing. Scale bar = 10 μm.

In sections stained with the pan-AMPAr antibody together with combinations of subunit-specific antibodies, we found that all of the puncta that were immunoreactive for GluR1, 2, 3 or 4 were also pan-AMPAr positive (Fig. 3). We therefore used the pan-AMPAr antibody to identify synapses that contained AMPArs and to determine the proportion of these synapses in each lamina that were labelled with the GluR1, 3 and 4 antibodies. For each of these subunits, 100 pan-AMPAr-positive puncta were analysed from each of laminae I, IIo, IIi and III in sections from 4 rats (Table 1). We analysed the two halves of lamina II separately, since there are differences in the main types of primary afferent input to each half, as well as in the response properties of their neurons [3]. We found that approximately 65% of the puncta in laminae I–II were GluR1-immunoreactive, while this proportion dropped to 34% in lamina III. Between 57–65% of puncta in laminae I–II were GluR3-positive, rising to 80% in lamina III. In contrast, only a small minority of puncta in the superficial laminae (23% in lamina I, 7–9% in lamina II) were positive with the GluR4-C antibody, and the proportion for lamina III was 46%. As expected [33] we found that virtually all pan-AMPAr-positive puncta were GluR2-immunoreactive in each of the laminae examined (Fig. 3e–h), and we therefore analysed a sample of 100 pan-AMPAr puncta located throughout the dorsoventral extent of laminae I–III in each rat. Between 98–100% (mean 99%) of these puncta were positive for GluR2 in sections from the 4 rats.

**Table 1:** Percentages of pAMPAr-positive puncta with different AMPAr subunits. Percentage of AMPAr-positive puncta in each lamina that were immunopositive with GluR1, GluR3 and GluR4-C antibodies. Each represents the mean value from 4 rats with the range given in brackets.

	**subunit**		
**Lamina**	**GluR1**	**GluR3**	**GluR4**

**I**	**64 **(59–68)	**65 **(54–70)	**23 **(15–34)
**IIo**	**66 **(58–70)	**57 **(54–63)	**7 **(3–10)
**IIi**	**63 **(57–70)	**57 **(53–59)	**9 **(5–11)
**III**	**34 **(27–42)	**80 **(76–86)	**46 **(38–54)

Since the quantification of GluR1 and GluR3 was carried out on the same set of sections, we were able to analyse the co-localisation of these two subunits. We found that the great majority (89–92%) of pan-AMPAr-positive puncta throughout laminae I–III were stained with either GluR1 or GluR3, while 25–37% contained both types of immunoreactivity (Fig. 3a–d, Table 2). However, most of the puncta that were strongly immunoreactive for GluR1 were either weakly stained or unstained with the GluR3 antibody, and *vice versa*. Approximately 10% of the pan-AMPAr-positive puncta in laminae I–III in these sections were not immunostained with either GluR1 or GluR3 antibodies (Table 2).

**Table 2:** Co-localization of GluR1 and GluR3. Percentage of AMPAr-positive puncta in each lamina that showed different patterns of immunoreactivity for GluR1 and GluR3. Each represents the mean value from 4 rats with the range given in brackets.

**Lamina**	**GluR1+/GluR3+**	**GluR1+/GluR3-**	**GluR1-/GluR3+**	**GluR1-/GluR3-**
**I**	**37 **(32–44)	**27 **(24–35)	**28 **(22–33)	**8 **(6–11)
**IIo**	**32 **(27–36)	**34 **(25–41)	**25 **(20–30)	**9 **(5–12)
**IIi**	**31 **(26–35)	**32 **(28–35)	**26 **(22–29)	**11 **(8–16)
**III**	**25 **(21–28)	**9 **(4–14)	**56 **(49–65)	**11 **(6–17)

**Figure 4:**
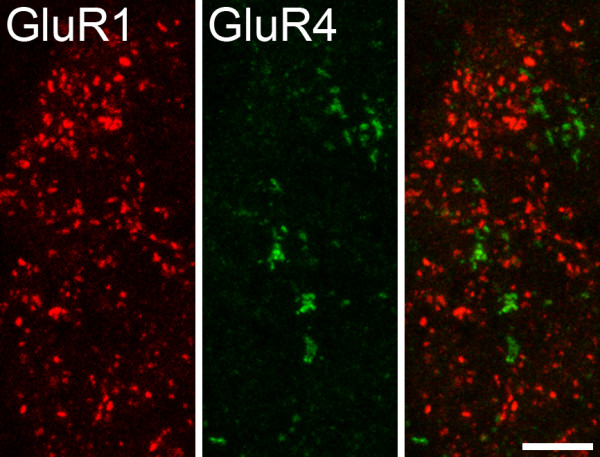
**Immunostaining for GluR1 and GluR4 in lamina I**. Confocal images that show immunoreactivity for GluR1 and GluR4, together with a merged image (right). Note that in this field the two types of immunoreactivity are contained in different puncta, with no co-localisation. This is a projection of 16 optical sections at 0.3 μm z-spacing. Scale bar = 5 μm.

Since both the GluR1 and GluR4-C antibody were raised in rabbit, we used a two-stage immunocytochemical procedure to look for possible co-existence between these subunits in lamina I. This region was chosen since it has a significant population of puncta that are immunoreactive with each of these antibodies, and because it also contains a relatively high density of projection neurons (see below). We found that the great majority of GluR4-immunoreactive puncta in lamina I were not labelled by the GluR1 antibody (Fig. 4), although a small proportion (mean 3.9%, range 1.5–6.9%, n = 3) also showed GluR1-immunoreactivity.

**Figure 5:**
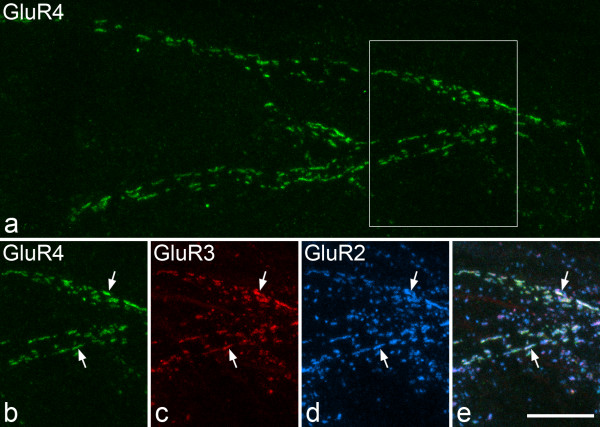
**Immunostaining for GluR2, GluR3 and GluR4 in lamina I**. **a**: A confocal image from a parasagittal section, showing a cluster of GluR4-immunoreactive puncta that appear to outline a single dendrite in lamina I. The boxed area is shown in **b**-**e**. **b**-**e**: part of the field shown in **a**, scanned to reveal GluR4, GluR3 and GluR2, together with a merged image. The GluR4-positive puncta are also GluR2- and GluR3-immunoreactive, and 2 of these are indicated with arrows. This is a projection of 19 optical sections at 0.3 μm z-spacing. Scale bar = 10 μm.

Although puncta immunoreactive with the GluR4-C antibody were relatively infrequent in laminae I–II, those that were present were often strongly immunoreactive and arranged in rows or clusters. Some of these were restricted to lamina I and were transversely (Fig. 1 arrow) or longitudinally orientated, while others that had a dorsoventral orientation were seen in laminae I, II or III (Fig. 1 arrowhead). Close inspection revealed that these clusters of bright puncta were frequently arranged in parallel rows which appeared to outline dendritic shafts (Fig. 5a). Those in lamina I were usually also strongly stained with the GluR3 antibody (Fig. 5b–c), while the dorsoventrally orientated ones in laminae II or III were generally either weakly stained or unstained with the GluR3 antibody (data not shown).

### AMPAr staining in KO mice

**Figure 6:**
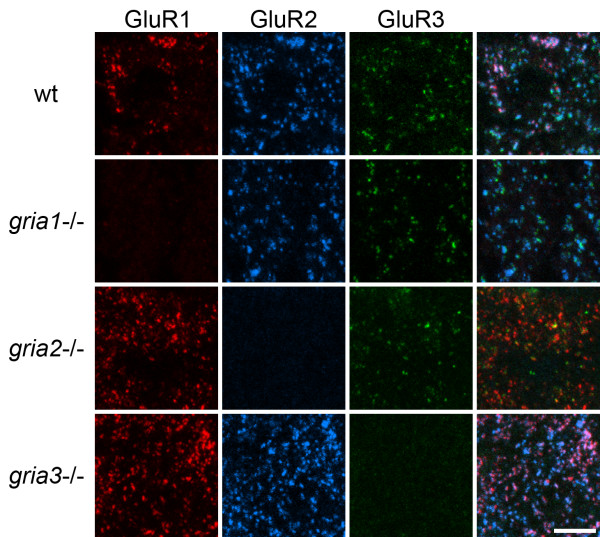
**Immunostaining for GluR1, GluR2 and GluR3 in AMPAr knock-out and wild-type mice**. Confocal images showing parts of the superficial dorsal horn from a wild-type (w.t.) mouse and from mice in which the genes coding for GluR1, GluR2 and GluR3 (g*ria1*, *2 *and *3*) had been knocked out. Each row shows staining in a different mouse, and in each case staining for GluR1 (red) is shown in the left column, followed by staining for GluR2 (blue) and GluR3 (green), with a merged image in the right column. Note the lack of staining for the corresponding subunit in each of the knock-out mice. Images show projections of 8 optical sections at 0.3 μm z-separation. Scale bar = 5 μm.

Sections from mice in which the GluR1-3 subunits had been knocked out and from corresponding wild-type animals were stained to reveal GluR1, GluR2 and GluR3, and the results are illustrated in Fig. 6. Sections from wild-type mice showed punctate staining with each of the 3 antibodies, and the laminar distribution resembled that seen in the rat dorsal horn. In each of the knock-outs staining with the antibody corresponding to the product of the deleted gene was completely absent, while an apparently normal pattern of punctate staining was detected with the other two antibodies.

### PSD-95 and its relation to pan-AMPAr staining

**Figure 7:**
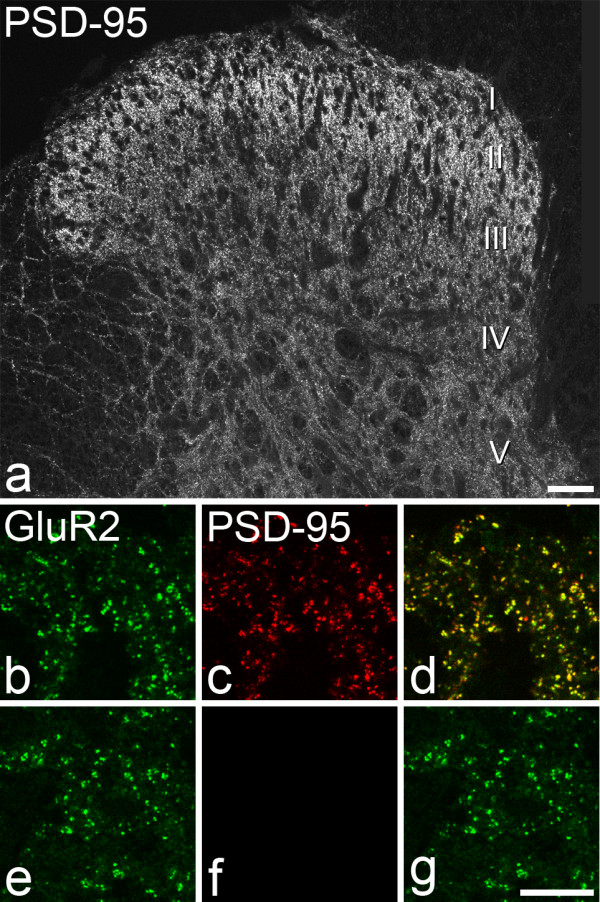
**PSD-95 immunostaining in dorsal horn**. **a**: The distribution of PSD-95-immunoreactivity in the rat dorsal horn following antigen retrieval with pepsin. Approximate positions of the laminae are shown. Punctate staining for PSD-95 is present throughout the dorsal horn, with the highest density in the superficial part (laminae I–II). **b**-**g **show immunostaining for GluR2 (green) and PSD-95 (red) in lamina II in a wild-type mouse (**b**-**d**) and in a PSD-95 mutant mouse (**e**-**g**). Note the lack of punctate staining for PSD-95 in the PSD-95 mutant. All images are from single optical sections. Scale bars = 50 μm (**a**), 10 μm (**b**-**g**).

Following antigen retrieval with pepsin, the PSD-95 antibody gave a punctate staining pattern in the rat dorsal horn, which was similar to that seen with the pan-AMPAr and GluR2 antibodies (Fig. 7a). Puncta were present throughout the grey matter of the spinal cord, but were densest in the superficial dorsal horn (laminae I–II). In sections from PSD-95-mutant (but not wild-type) mice, staining with the PSD-95 antibody was absent, while staining with GluR2 antibody in the same sections was apparently normal (Fig. 7b–g).

**Figure 8:**
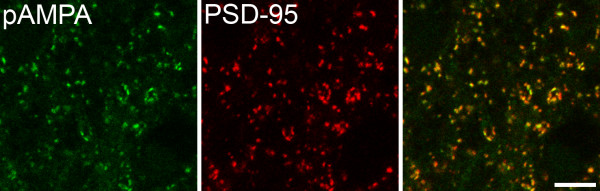
**Co-localisation of PSD-95 and pan-AMPAr in lamina II**. Confocal images showing the co-localisation of immunostaining for pan-AMPAr (pAMPA) and PSD-95 in lamina IIi. These images were projected from 2 optical sections at 0.3 μm z-separation. Scale bar = 5 μm.

In each of 3 rats, we analysed 100 pan-AMPAr-immunoreactive puncta from laminae I–III and found that virtually all of them (mean 99.7%, range 99–100) were also positive for PSD-95 (Fig. 8). We also found that 97.7% (96–99%) of PSD-95-immunoreactive puncta were labelled with the pan-AMPAr antibody.

## Discussion

The main novel findings of this study are (1) that GluR1 is present at the majority of AMPAr-containing synapses (~65%) in laminae I–II, where it is invariably co-localised with GluR2 and often with GluR3, and (2) that antibody against the C-terminal part of GluR4 labels relatively few synapses in the superficial laminae, with those in lamina I being largely separate from GluR1-containing puncta. We confirm that virtually all AMPAr-containing synapses in the superficial dorsal horn possess GluR2 subunits. We also provide further evidence that the staining seen with AMPAr antibodies after antigen retrieval corresponds to synaptic receptors, by demonstrating a high degree of co-localisation with the postsynaptic density protein PSD-95, and by showing that punctate staining with GluR1-3 antibodies is absent in tissue from corresponding knock-out mice.

### PSD-95 in the dorsal horn

PSD-95, a member of the membrane-associated guanylate kinase (MAGUK) family, is a major constituent of the post-synaptic density at glutamatergic synapses, and contains domains that are involved in protein-protein interactions. PSD-95 is known to play an important role in NMDA receptor-mediated synaptic plasticity [43-45], and in the spinal cord it appears to be necessary for the development of neuropathic pain following peripheral nerve injury [46,47], although not for inflammatory pain [47]. There have apparently been few studies of the distribution of PSD-95 in the spinal cord, although Tao et al. [48] used an immunocytochemical method without antigen retrieval and reported staining that was densest in laminae I and IIo, while Garry et al. [47] observed β-galactosidase activity associated with PSD-95 expression that was restricted to neurons in lamina II. Fukaya and Watanabe [40] demonstrated that treatment of sections with pepsin prior to immunocytochemistry unmasks epitopes and allows detection of MAGUK proteins in the post-synaptic density, whereas these were not visualised reliably with conventional methods. It is therefore likely that the punctate pattern that we have demonstrated corresponds to the distribution of PSD-95 in the post-synaptic densities of glutamatergic synapses, and the finding that this staining was absent in PSD-95 mutant mice confirms that the antibody is specific for the protein. Interestingly, the antibody that we used was raised against the N terminal portion of PSD-95, which is intact in the mutant mouse. Migaud et al. [43] demonstrated that a truncated form of the protein, known as PSD^PDZ12^, was synthesised in these animals. However, they concluded that this was not localised to post-synaptic densities, and our results confirm this interpretation.

The almost perfect co-localisation of PSD-95- and pan-AMPAr-immunostaining seen in the present study strongly suggests that both proteins are expressed at virtually all glutamatergic synapses in the superficial dorsal horn in the adult rat. Although it is possible that there is a population of glutamatergic synapses that lack both proteins, there is apparently no evidence to support such a suggestion.

### Comparison with previous findings

Although we did not determine the percentage of glutamatergic synapses that expressed GluR1 in our previous study [33] we did estimate that this subunit was present at 30–40% of synapses associated with terminals that contained VGLUT1 or were derived from unmyelinated primary afferents in laminae I–II, and at ~15% of the synapses formed by VGLUT2-containing boutons in these laminae. However, in the present study we found that ~65% of puncta in these laminae were GluR1-immunoreactive. In both the quantitative analysis of GluR1 in our previous study [33] and also in the present study, we used the same GluR1 antibody and detected this with a Cy5-conjugated secondary antibody. However, in the present study the Cy5 fluorescence was revealed with a highly sensitive gallium arsenide phosphide (GaAsP) PMT, and the improved sensitivity probably accounts for the much higher expression that we observed here.

In contrast, we found that only 7–23% of puncta in laminae I and II were immunoreactive with an antibody against the C-terminal portion of GluR4, whereas in our previous study we reported that more than 40% of puncta in these laminae were stained with the GluR4-N antibody [33]. This discrepancy resulted from the relatively weak staining by the GluR4-N antibody of many puncta that were negative with the GluR4-C antibody, as shown in Fig. 2. There are a number of possible explanations for the difference in staining patterns between the two antibodies. Firstly, the GluR4-N antibody may cross-react with another protein, for example one of the other AMPAr subunits. This seems very unlikely since in the hippocampus staining with this antibody is restricted to the dendrites of interneurons, and is not seen on pyramidal cells, which express GluR1, 2 and 3 subunits (M.W. unpublished observations). Alternatively, it may be that immunostaining with the GluR4-N antibody is more sensitive, resulting in identification of a population of puncta that are below the detection threshold with the GluR4-C antibody. However, we found that the puncta that are positive with the GluR4-C antibody in laminae I–II were often very strongly labelled, which argues against this explanation. Nonetheless, it is possible that the difference in location of the epitopes (extracellular for GluR4-N, intracellular for GluR4-C) is a contributory factor. A third explanation is that the alternatively spliced short form of GluR4 (GluR4c) [49], which lacks the epitope recognised by the GluR4-C antibody, is expressed at some synapses in the superficial dorsal horn that have undetectable levels of the long form of GluR4. As far as we are aware, there is little evidence available concerning the expression of GluR4c in the spinal cord, although Kawahara et al. [50] have estimated that approximately 10% of the mRNA for GluR4 in adult human spinal grey matter is the GluR4c form. Whatever the explanation, the present findings suggest that the long form of GluR4 is highly expressed in only a small proportion of synapses in the superficial dorsal horn.

### AMPAr subunit expression at synapses in the superficial dorsal horn

Our results suggest that the great majority of cells with dendrites in laminae I and II express GluR2 together with either or both of the GluR1 and GluR3 subunits (and possibly low levels of GluR4c). Approximately 10% of puncta were found to lack both GluR1- and GluR3-immunoreactivity, and these may correspond to those that express the GluR4 subunit. Alternatively, it may be that these synapses do contain GluR1 or GluR3, but at levels that were below the detection threshold with the methods used in this study.

The finding that ~65% of puncta in laminae I–II were GluR1-immunoreactive suggests that this subunit is expressed by the majority of cells in these laminae. There are several lines of evidence to suggest that this subunit plays a role in synaptic plasticity in the dorsal horn in pain states. Zhou et al. [51] observed a rapid up-regulation of GluR1 mRNA in the lumbar dorsal horn following injection of complete Freund's adjuvant into the hindpaw, while Fang et al. [52] reported phosphorylation of this subunit at both S831 and S845 sites in Western blots of spinal cord tissue after intradermal capsaicin injection. We were able to demonstrate S845 phosphorylation of GluR1 at synapses in laminae I–II of the ipsilateral dorsal horn after capsaicin injection [33]. In addition, it has been reported that GluR1-containing receptors can be recruited to neuronal plasma membranes in lumbar spinal cord following a noxious visceral stimulus [53]. Synaptic plasticity involving GluR1 could therefore potentially occur in a high proportion of neurons in the superficial dorsal horn.

Interestingly, the GluR4-C-immunoreactive puncta seen in laminae I and II were often arranged in clusters, and in fortuitous sections these appeared to outline parts of the dendritic trees of individual neurons, as shown in Fig. 5. These neurons are presumably relatively infrequent (since only a few clusters were observed in each section) and have a high density of glutamatergic synapses on their dendritic trees. The arrangement of puncta indicated that the dendrites were often of relatively large diameter. The clusters of GluR4-immunoreactive puncta seen in lamina I may belong to projection cells, which are thought to make up approximately 5% of the neuronal population in this lamina [54]. Projection cells with the neurokinin 1 (NK1) receptor have been shown to have a high density of synaptic input from substance P-containing primary afferents (which are also glutamatergic) [12], and we have recently observed that a population of large projection cells that lack the NK1 receptor [55] receive numerous contacts from VGLUT2-immunoreactive boutons on their dendritic trees (AJT and EP, unpublished observations).

Dorsoventrally-orientated clusters of GluR4-C-immunoreactive puncta were also seen in laminae II and III, and these may represent either the ventrally directed dendrites of lamina I cells or else dorsal dendrites of cells located in deeper laminae. Projection neurons in laminae III and IV that express the NK1 receptor have been shown to have dorsally directed dendrites that extend into lamina I [6], and these are known to have a high density of synaptic input from substance P-containing afferents [56]. It is therefore possible that some of the clusters of GluR4-immunoreactive puncta are associated with these cells.

Since the majority of glutamatergic synapses in lamina I appear to contain GluR1, it is likely that some of the projection neurons in this lamina express this subunit. However, these would belong to a different population to those that express GluR4, since the 2 subunits were very seldom colocalised. It has been shown that lamina I projection neurons can develop LTP following either high or low-frequency stimulation of primary afferents [57,58]. Activity-dependent insertion of AMPArs that contain either GluR1 or GluR4 is thought to underlie certain forms of LTP, and it is therefore likely that either GluR1- or GluR4-containing receptors are responsible for this phenomenon in lamina I projection cells.

## Conclusion

Our results provide further confirmation that the immunostaining seen in the spinal dorsal horn with antibodies against AMPArs, following antigen retrieval with pepsin, represents receptors located at glutamatergic synapses. They show that the majority of such synapses in laminae I–III contain GluR2 with either or both of GluR1 and GluR3 subunits. In contrast, the long form of the GluR4 subunit has a much more restricted distribution in the superficial dorsal horn, and appears to be associated with specific types of neuron, many of which also express GluR2 and GluR3, but not GluR1. These neurons may correspond, at least in part, to lamina I projection cells. The high degree of co-localisation of pan-AMPAr staining with that for PSD-95 suggests that virtually all glutamatergic synapses in this region contain AMPArs in the adult spinal cord.

## Methods

### Animals

Eighteen adult male Wistar rats (Harlan, Loughborough, UK; 230 – 300 g) were deeply anaesthetised with pentobarbitone and perfused through the left ventricle with Ringer's solution followed by 4% freshly depolymerised formaldehyde. Lumbar segments (L2–L5) were removed, stored in the same fixative for 5–8 hours and then cut into transverse or parasagittal 60 μm thick sections with a Vibratome. In addition, spinal cord tissue that had been fixed according to the same protocol was obtained from adult mice of either sex that lacked the genes for GluR1 (*gria1*-/-), GluR2 (*gria2*-/-) or GluR3 (*gria3*-/-), or had a mutation affecting the gene coding for PSD-95 [43], together with tissue from corresponding adult wild-type mice. The mouse spinal cords were cut with a Vibratome into transverse 60 μm thick sections. Sections were rinsed for 30 mins in 50% ethanol to enhance antibody penetration.

All experiments were approved by the Ethical Review Process Applications Panel of the University of Glasgow, and were performed in accordance with the European Community directive 86/609/EC and the UK Animals (Scientific Procedures) Act 1986. All efforts were made to minimize the number of animals used and their suffering.

### Immunocytochemistry

All sections underwent antigen retrieval with pepsin prior to immunocytochemical processing [33,38]. This involved incubating the sections at 37°C for 30 mins in PBS followed by 10 mins in 0.2 M HCl containing 1 mg/ml pepsin (Dako, Glostrup, Denmark). They were then reacted for double- or triple-immunofluorescence labelling with various combinations of antibodies directed against AMPAr subunits or PSD-95 (see Table 3 for details of the antibodies used and their concentrations). In most cases, sections were incubated for 2–3 days at 4°C in a cocktail of 2 or 3 primary antibodies (each raised in a different species), and then overnight at 4°C in species-specific secondary antibodies that were raised in donkey and conjugated to either Alexa 488 (Invitrogen, Paisley, UK; 1:500), or to Rhodamine Red or Cy5 (Jackson Immunoresearch, West Grove, PA, USA; 1:100). For the sections that were reacted with both GluR1 and GluR4-C antibodies a different approach was used, since both of these antibodies were raised in rabbit. In this case, sections were initially incubated for 1 day in GluR1 antibody followed by 1 day in Fab' fragment of donkey anti-rabbit IgG conjugated to Rhodamine Red (Jackson Immunoresearch; 1:100). They were then incubated in unlabelled Fab' fragment of donkey anti-rabbit IgG (Jackson Immunoresearch; 1:20) for 2 hours (to block any binding sites on the GluR1 antibody), followed by 3 days in rabbit anti-GluR4-C and 1 day in Alexa 488-labelled donkey anti-rabbit IgG (Invitrogen; 1:500).

**Table 3:** Characteristics of primary antibodies used in this study

**Antibody**	**Source**	**Species, Type**	**Dilution**	**Reference**
GluR1	Chemicon	Rabbit, polyclonal	1:500	
GluR2	Chemicon	Mouse, monoclonal	1:300	[59]
GluR3	M. Watanabe	Goat, polyclonal	1:500	
GluR4-N	M. Watanabe	Guinea pig, polyclonal	1:500	[33]
GluR4-C	LabVision	Rabbit, polyclonal	1:100	
pan-AMPAr	M. Watanabe	Guinea pig, polyclonal	1:100	[39]
PSD-95	M. Watanabe	Rabbit, polyclonal	1:200	[40]

For all immunocytochemical reaction, the rinses were in PBS with 0.3 M NaCl, and antibodies were diluted in PBS that contained 0.3% Triton-X100. Sections were mounted in anti-fade medium (Vectashield; Vector Laboratories, Peterborough, UK) and stored at -20°C.

### Antibodies

The goat GluR3 antibody was raised against glutathione *S*-transferase fused to the C terminal residues 830–862 of the mouse GluR3 subunit (GenBank accession number AB022342) and affinity-purified using GST fusion protein-coupled cyanogen bromide-activated Sepharose 4B (Amersham Biosciences, Bucks, UK) as described previously [33].

All of the other polyclonal antibodies used in this study were also affinity-purified. The GluR1 antibody (Chemicon, Chandlers Ford, UK; cat no. AB1504) was raised against a synthetic peptide corresponding to the last 13 amino acids of rat GluR1 and is reported to show no cross-reactivity with other AMPAr subunits (manufacturer's specification), while the monoclonal GluR2 antibody (Chemicon, cat no. mab397, clone 6C4) has been extensively characterised and shown not to detect other AMPA or kainate subunits [59]. The GluR4-N antibody was raised against residues 245–273 of mouse GluR4 and recognises a single protein band of ~98 kDa in Western blots of the PSD fraction from mouse spinal cord [33]. Staining with this antibody is blocked by pre-incubation with the immunising peptide [33]. The GluR4-C antibody (Labvision, Fremont, CA, USA; cat no. RB-9059) was raised against a peptide derived from the C-terminal of human GluR4 and recognises a single band of ~105 kDa in Western blots of rat brain lysates (manufacturer's specification). The PSD-95 antibody was raised against the N-terminal region (residues 1–64) of mouse PSD-95 and recognised a band of 87–97 kDa on Western blots of rat brain homogenates [40]. Finally, the pan-AMPAr antibody was raised against residues 717–745 of the mouse GluR1 (a region that shows high sequence homology between the GluR1-GluR4 subunits) and detected each of the four AMPAr subunits in transfected cells, with a trace of cross-reactivity to the kainate receptor subunit GluR6 [39].

### Confocal microscopy and Analysis

Sections were scanned with a Bio-Rad Radiance 2100 confocal microscope with Argon, HeNe and red diode lasers, or a Bio-Rad MRC1024 confocal with a Krypton-Argon laser. All of the analysis was carried out on stacks of confocal images scanned sequentially (to avoid fluorescent bleed-through) with a 60× oil-immersion lens and a z-separation of 0.3 or 0.35 μm.

Analysis of the percentage of AMPAr puncta that were immunolabelled for GluR1, GluR3 or GluR4 was carried out on sections from 4 rats. From each rat, one section that had been reacted with pan-AMPAr, GluR1 and GluR3 antibodies, and one that had been reacted with pan-AMPAr and GluR4-C antibodies was selected. From each section, a set of confocal scans covering a ~100 μm wide strip through the entire dorsoventral extent of laminae I–III was obtained. The lamina I/II and II/III borders were identified by examining sections through a dark-field condenser [60], while the lamina III/IV border was determined by reference to an atlas of rat spinal cord [61]. Lamina II was divided into outer (IIo) and inner (IIi) parts by drawing a line midway between the I/II and II/III borders. pan-AMPAr-immunostaining in confocal scans was examined with MetaMorph software (Universal Imaging, Downington, PA, USA) and immunoreactive puncta were initially selected by using a grid [33]. This was done in such a way as to ensure that puncta through the full dorsoventral extent of each lamina were selected. From each section, 400 pan-AMPAr-labelled puncta were selected (100 each from laminae I, IIo, IIi and III) and these were then examined to determine whether they were immunoreactive with the GluR1, GluR3 or GluR4 antibodies.

Examination of sections that had been reacted with pan-AMPAr antibody and GluR2 revealed an almost perfect co-localisation throughout laminae I–III. We therefore used a modification of the approach described above, in which 100 pan-AMPAr puncta were selected from the full dorsoventral extent of this region in a single section each from 4 rats and examined for the presence of GluR2.

To determine the extent of GluR1 and GluR4 co-localisation in lamina I, confocal scans were obtained from 4 sections each from 3 rats. The confocal images representing GluR4 were initially examined with Neurolucida for Confocal (MicroBrightField Inc., Colchester, VT, USA) and at least 25 GluR4-immunoreactive puncta were selected from each section. Confocal images representing both types of immunoreactivity were then merged and the presence or absence of GluR1-immunostaining in each of the selected puncta was recorded.

To investigate co-localisation of AMPArs with PSD-95, one section that had been reacted with pan-AMPAr and PSD-95 antibodies was selected from each of 3 rats. In each case a set of confocal images was scanned to produce a ~100 μm-wide strip through the full extent of laminae I–III. One hundred PSD-95-immunoreactive puncta were initially selected from the full dorsoventral extent of this region and examined to determine whether they were also pan-AMPAr-immunoreactive. A similar approach was then used to determine the proportion of pan-AMPAr-immunoreactive puncta that were labelled with the PSD-95 antibody. In each case, the selection of puncta was made while the observer was blind to the other type of immunostaining.

### Immunostaining of mouse tissue

Sections from *gria1*-/-, *gria2*-/- and *gria3*-/- mice (n = 2 for each mutation) and appropriate wild-type animals were reacted with antibodies against GluR1, GluR2 and GluR3, while those from 3 PSD-95-mutant and wild-type mice were reacted with PSD-95 and GluR2 antibodies.

## Competing interests

The author(s) declare that they have no competing interests.

## Authors' contributions

EP participated in the design of the study and the analysis; MW generated several of the antibodies; BH participated in some of the experiments; SGNG generated the PSD-95 mutant mice; AJT conceived of the study, participated in design and analysis and drafted the manuscript. All authors participated in the writing of the manuscript and approved the final version.
